# Erinacine A Prevents Lipopolysaccharide-Mediated Glial Cell Activation to Protect Dopaminergic Neurons against Inflammatory Factor-Induced Cell Death In Vitro and In Vivo

**DOI:** 10.3390/ijms23020810

**Published:** 2022-01-12

**Authors:** Shou-Lun Lee, Jing-Ya Hsu, Ting-Chun Chen, Chun-Chih Huang, Tzong-Yuan Wu, Ting-Yu Chin

**Affiliations:** 1 Department of Biological Science and Technology, China Medical University, Taichung 406040, Taiwan; 2 Department of Bioscience Technology, Chung Yuan Christian University, Taoyuan 320314, Taiwan; kyoya05050815@gmail.com (J.-Y.H.); tingchun107@gmail.com (T.-C.C.); 3 New Bellus Enterprise Co., Ltd., Tainan 720008, Taiwan; john@newbellus.com.tw; 4 Department of Chemistry, Chung Yuan Christian University, Taoyuan 320314, Taiwan; 5 Center for Nano Technology, Chung Yuan Christian University, Taoyuan 320314, Taiwan

**Keywords:** erinacine A, neuroprotection, neuroinflammation, lipopolysaccharide, Parkinson’s disease

## Abstract

*Hericium erinaceus* (HE) is a common edible mushroom consumed in several Asian countries and considered to be a medicinal mushroom with neuroprotective effects. Erinacine A (EA) is a bioactive compound in *Hericium erinaceus* mycelium (HEM) that has been shown to have a neuroprotective effect against neurodegenerative diseases, e.g., Parkinson’s disease (PD). Although the etiology of PD is still unclear, neuroinflammation may play an important role in causing dopaminergic neuron loss, which is a pathological hallmark of PD. However, glial cell activation has a close relationship with neuroinflammation. Thus, this study aimed to investigate the anti-neuroinflammatory and neuroprotective effects of EA on lipopolysaccharide (LPS)-induced glial cell activation and neural damage in vitro and in vivo. For the in vitro experiments, glial cells, BV-2 microglial cells and CTX TNA2 astrocytes were pretreated with EA and then stimulated with LPS and/or IFN-γ. The expression of proinflammatory factors in the cells and culture medium was analyzed. In addition, differentiated neuro-2a (N2a) cells were pretreated with EA or HEM and then stimulated with LPS-treated BV-2 conditioned medium (CM). The cell viability and the amount of tyrosine hydroxylase (TH) and mitogen-activated protein kinases (MAPKs) were analyzed. In vivo, rats were given EA or HEM by oral gavage prior to injection of LPS into the substantia nigra (SN). Motor coordination of the rats and the expression of proinflammatory mediators in the midbrain were analyzed. EA pretreatment prevented LPS-induced iNOS expression and NO production in BV-2 cells and TNF-α expression in CTX TNA2 cells. In addition, both EA and HEM pretreatment significantly increased cell viability and TH expression and suppressed the phosphorylation of JNK and NF- κB in differentiated N2a cells treated with CM. In vivo, both EA and HEM significantly improved motor dysfunction in the rotarod test and the amphetamine-induced rotation test and reduced the expression of TNF-α, IL-1β and iNOS in the midbrain of rats intranigrally injected with LPS. The results demonstrate that EA ameliorates LPS-induced neuroinflammation and has neuroprotective properties.

## 1. Introduction

Communication between neurons and glial cells is fundamental to maintain the parenchymal structure and function in the brain. Immune responses in the central nervous system (CNS) are mediated by microglia and astrocytes, which are protective and restorative responses to CNS infection or injury [1]. Both microglia and astrocytes are primarily involved in neuroinflammation and display proinflammatory or anti-inflammatory phenotypes that are affected by the invading stimuli and the microenvironment [2]. The proinflammatory phenotype of activated microglia releases proinflammatory molecules such as TNF-α, interleukin (IL)-1β, IL-6, and reactive oxygen/nitrogen species [3]. Likewise, BV-2 mouse microglial cells stimulated with LPS and IFN-γ resulted in increased release of both NO and proinflammatory cytokine expression. Activated microglia release mediators that induce the activation of astrocytes and modulate the function of astrocytes [2]. Li et al. found that LPS induces proinflammatory activation of astrocytes [4]. Multiple studies have demonstrated that LPS induces TNF-α, IL-1β, and NO in astrocytes [5].

The proinflammatory phenotype of glial cells may have a harmful effect on the brain and contribute to the susceptibility to and/or the progression of neurodegenerative diseases, e.g., Parkinson’s disease (PD) [6]. The neuronal aggregation of α-synuclein inclusions and the loss of dopaminergic neurons in the substantia nigra pars compacta (SNpc) are pathological hallmarks of PD. Several reports have demonstrated that α-synuclein induces both astrocyte and microglial activation and the release of proinflammatory factors [7,8,9]. The overexpression of α-synuclein in astrocytes can increase the expression of inflammatory cytokines and chemokines, e.g., IFN-γ and TNF-α [10,11]. In addition, α-synuclein accumulated in astrocytes can produce various oxygen-free radicals and cytotoxic factors, e.g., IL-1β and TNF-α, resulting in injury and apoptosis of dopaminergic neurons [12]. Moreover, studies have suggested that inflammation may drive the progressive loss of SN dopaminergic neurons in vivo [13]. Therefore, modulation of neuroinflammation is an important issue in the prevention and treatment of PD.

*Hericium erinaceus* (HE), also known as Lion Mane mushroom, Monkey Head mushroom, Satyr’s beard and Yamabushitake, is a common mushroom consumed in several Asian countries and considered to be a medicinal mushroom with immunomodulatory, antibacterial, anticancer, hypertension and hyperlipidemia and neuroprotective effects [14]. Many bioactive compounds with medicinal properties have been isolated from HE and identified, e.g., erinacines and hericenones [15]. Both erinacines and hericenones can pass through the blood-brain barrier to the brain and they possess neuroprotective effects [16,17]. Erinacine A (EA) is the main component of the erinacines [15] and it has been shown to have neuroprotective effects against depressive disorder [18], ischemic injury [19], Alzheimer’s disease [20,21] and Parkinson’s disease in vivo [22]. However, no studies have elucidated the effect of EA on glial cells.

Currently, no age-related neurodegenerative diseases are curable. The prevention of neurodegeneration is an important research topic. The purpose of this study was to explore the mechanisms of how EA-pretreated glial cells resist LPS-induced inflammation in vitro and in vivo. We found that EA selectively modulates the expression of proinflammatory genes in microglia and astrocytes and prevents LPS-induced neural death. Both in vitro and in vivo studies found that EA modulates neuroinflammation and possesses neuroprotective activity.

## 2. Results

### 2.1. EA Selectively Modulates the Expression of Proinflammatory Genes in Glia

**Figure 1:**
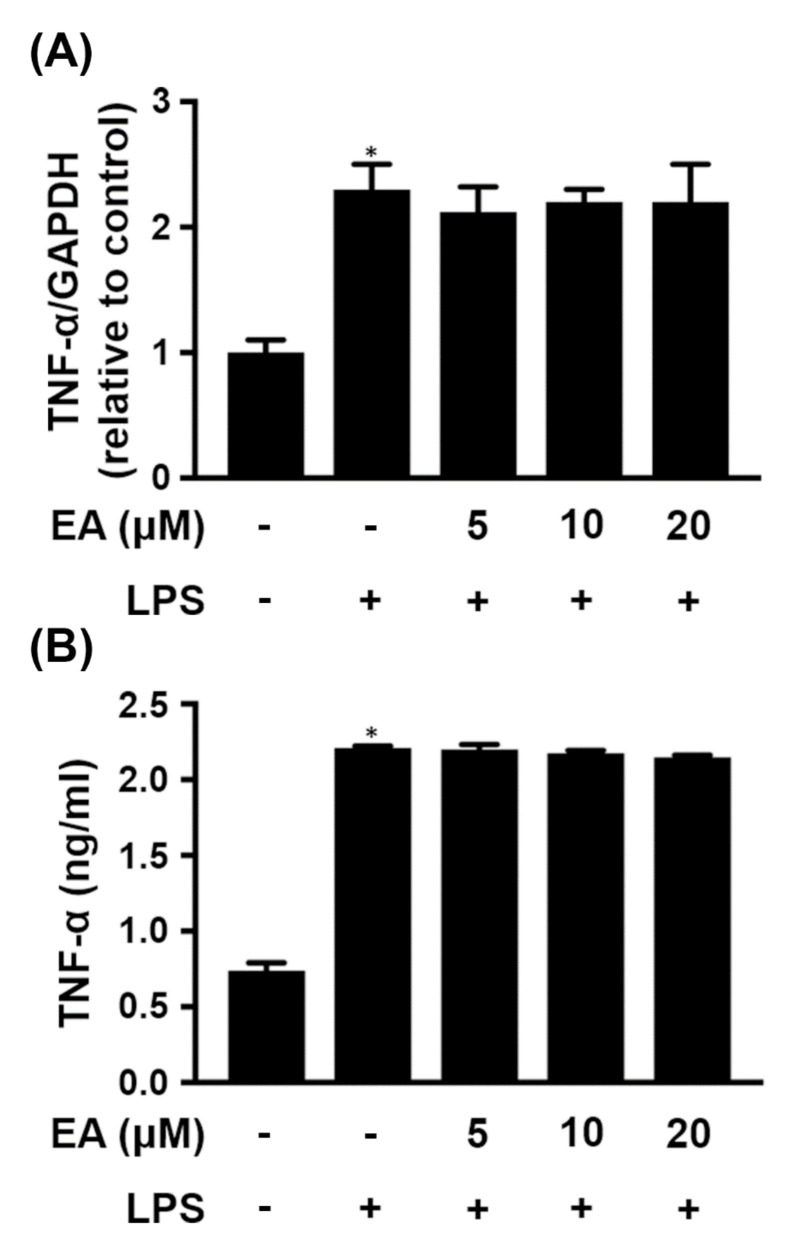
Effect of EA on TNF-α expression in LPS-treated BV-2 cells. BV-2 cells were treated with the indicated concentration of EA for 15 min and then stimulated with LPS (250 ng/mL) for 24 h. (**A**) The transcriptional levels of TNF-α were detected in BV-2 cells by qRT-PCR (*n* = 3). (**B**) The protein levels of TNF-α in the culture medium were analyzed by ELISA (*n* = 3). * *p* < 0.05 compared to the untreated group.

Activated microglia release proinflammatory mediators that are implicated in neurodegenerative diseases [23]. Treatment of BV-2 cells, a microglial cell line, with LPS (250 ng/mL) significantly increased the expression of TNF-α, which was detected not only in the cells (Figure 1A) but also in the culture medium (Figure 1B). However, the expression of TNF-α was not affected whether cells were pretreated with or without EA before treatment with LPS (Figure 1).

**Figure 2:**
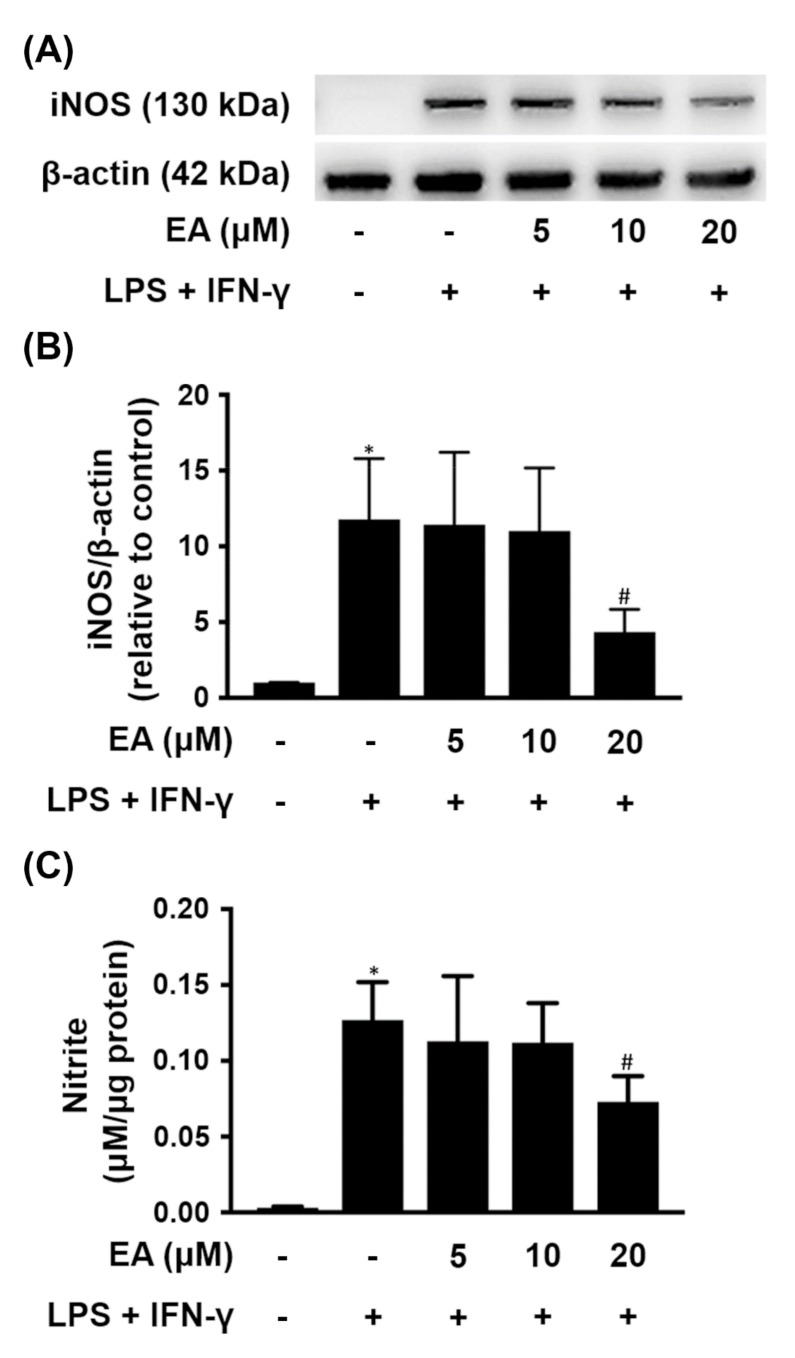
Effect of EA on iNOS expression and NO production in BV-2 cells treated with LPS and IFN-γ. BV-2 cells were treated with the indicated concentration of EA for 15 min and then stimulated with LPS (500 ng/mL) and IFN-γ (50 ng/mL) for 24 h. (**A**) The protein levels of iNOS were detected by western blot (*n* = 3). β-actin was used as a loading control. (**B**) Quantitative densitometry was used to calculate the ratio of iNOS to β-actin, which was normalized to the control. The relative density was estimated by NIH ImageJ densitometric analysis. (**C**) Estimated NO release derived from the nitrite present in the culture medium using the Griess reagent assay (*n* = 3). The results are expressed as the mean ± SD. * *p* < 0.05 compared to the untreated group. ^#^ *p* < 0.05 compared to the LPS/IFN-γ groups.

In addition, when BV-2 cells were treated with LPS (500 ng/mL) and IFN-γ (50 ng/mL), iNOS was overexpressed, contributing to significantly increased production of NO in the culture medium (Figure 2). Low concentrations of EA (5 µM or 10 µM) pretreatment did not reduce both iNOS expression and NO production in BV-2 cells treated with LPS and IFN-γ. However, BV-2 cells were pretreated with EA (20 µM) for 15 min before treatment with both LPS and IFN-γ, resulting in reduced iNOS expression and NO formation, which were significantly reduced by 59% and 43%, respectively, compared with cells cultured in the absence of EA (Figure 2B,C). Therefore, EA pretreatment prevented LPS-induced iNOS expression in BV-2 microglial cells.

**Figure 3:**
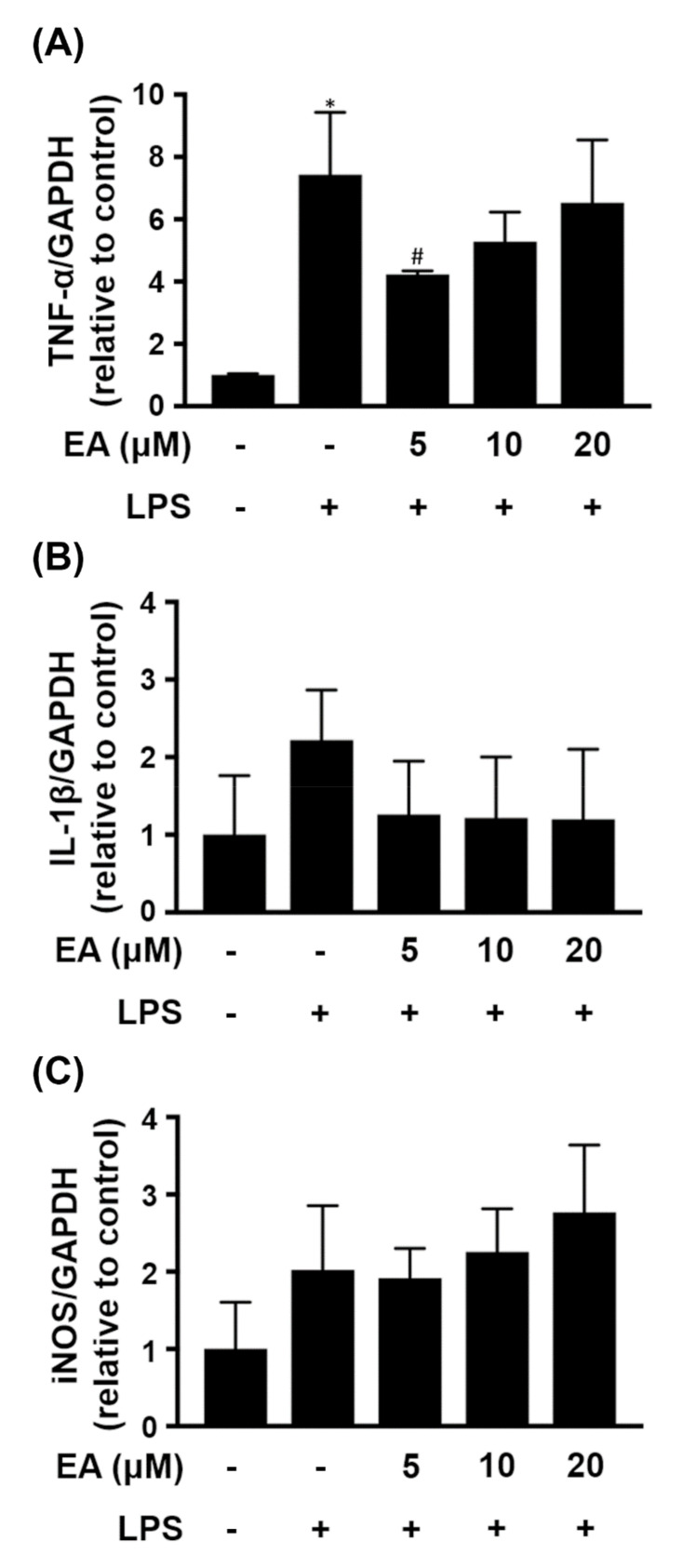
Effect of EA on proinflammatory mediators in LPS-treated CTX TNA2 cells. CTX TNA2 cells were treated with the indicated concentration of EA for 15 min and then stimulated with LPS (250 ng/mL) for 24 h. The transcriptional levels of (**A**) TNF-α, (**B**) IL-1β and (**C**) iNOS in the cells were detected by qRT-PCR (*n* = 3). The data are expressed as fold changes relative to the control rats. The results are expressed as the mean ± SD. * *p* < 0.05 compared to the control group, ^#^ *p* < 0.05 compared to the LPS groups.

Astrocytes represent the largest percentage of glia in the CNS and they participate in the inflammatory response in neurodegenerative diseases [4]. CTX TNA2 cells, an astrocyte cell line, were treated with LPS (250 ng/mL) for 24 h, resulting in significantly enhanced expression of TNF-α. The cells were pretreated with EA (5 µM) for 15 min before treatment with LPS for 24 h, and the expression of TNF-α was significantly reduced by 43% (Figure 3A). However, pretreatment with the concentration of EA more than 5 µM did not reduce the expression of TNF-α in CTX TNA2 cells treated with LPS. In addition, the expression levels of IL-1β and iNOS were not different after the cells were treated with EA or LPS (Figure 3B,C).

**Figure 4:**
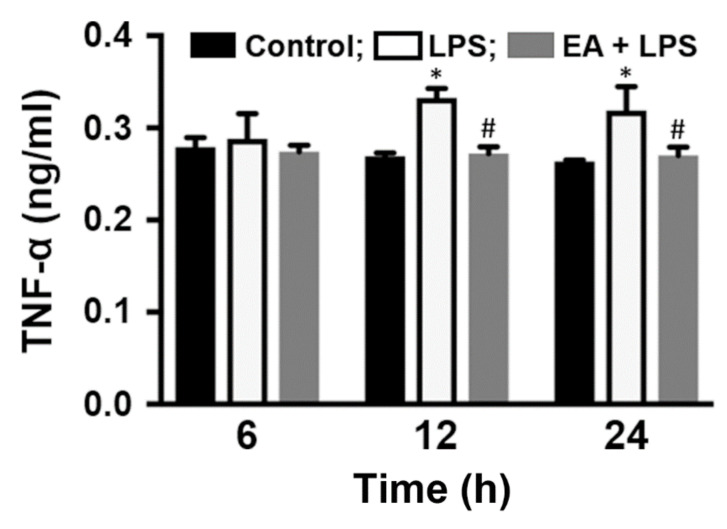
EA inhibits TNF-α production in CTX TNA2 cells. CTX TNA2 cells were treated with EA (10 µM) for 15 min and then stimulated with LPS (250 ng/mL) for 6, 12 or 24 h. The protein levels of TNF-α in the culture medium were analyzed by ELISA (*n* = 3). * *p* < 0.05 compared to the untreated group, ^#^ *p* < 0.05 compared to the LPS groups.

Furthermore, the level of TNF-α in the culture medium was measured. The level of TNF-α was not different after the cells were treated with EA or LPS for 6 h. However, CTX TNA2 cells were treated with LPS for 12 h and 24 h, and the level of TNF-α in the culture medium was significantly increased by 23% and 21%, respectively (Figure 4). Pretreatment with EA (10 µM) for 15 min and then stimulation with LPS for 12 h and 24 h reduced the level of TNF-α in the culture medium, which was significantly reduced by 18% and 15%, respectively, compared to that in cells without EA treatment (Figure 4). Therefore, EA pretreatment prevented LPS-induced TNF-α expression in CTX TNA2 cells.

### 2.2. HEM/EA Ameliorates Microglia-Induced Neurotoxicity

**Figure 5:**
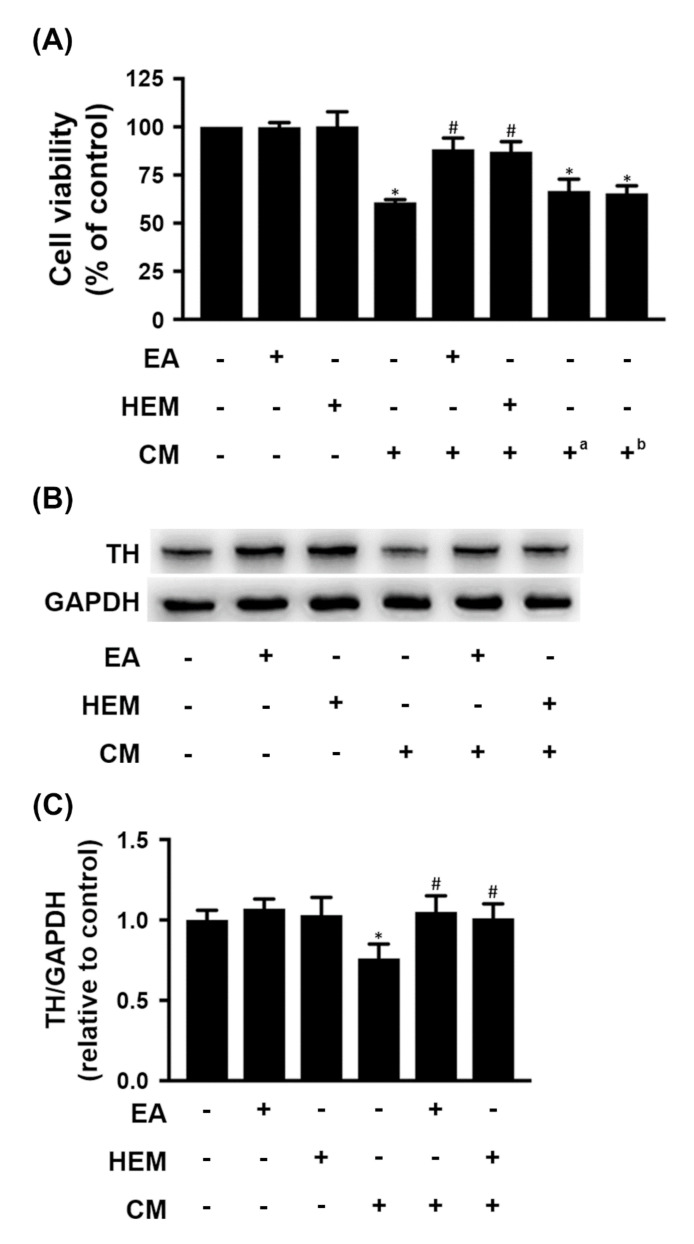
Effects of EA or HEM on microglia-mediated neurotoxicity. N2a cells after incubation in mixed differentiating conditions (0.5% FBS and 1 mM dbcAMP) for 5 days treated with or without EA (10 µM) or HEM (1 mg/mL) for 48 h or 24 h, half of the medium was removed, and the same amount of conditioned medium was added for 24 h. Conditioned medium (CM) was derived from the culture medium of BV-2 cells with 250 ng/mL LPS treatment for 24 h. (**A**) Cell viability of differentiated N2a cells was examined by MTS assay (*n* = 3). ^a^ CM contained 10 µM EA; ^b^ CM contained 1 mg/mL HEM. (**B**) The protein levels of TH were detected in N2a cells by western blot (*n* = 3). GAPDH was used as a loading control. (**C**) Quantitative densitometry was used to calculate the ratio of TH to GAPDH, which was normalized to the control. The relative density was estimated by NIH ImageJ densitometric analysis. The results are expressed as the mean ± SD. * *p* < 0.05 compared to the control group. ^#^ *p* < 0.05 compared to the BV-2 conditioned medium treatment group.

HEM and its bioactive compound EA have been revealed as potential neuroprotective agents. Herein, differentiated N2a cells were treated with HEM (1 mg/mL) or EA (10 µM) for 48 h, and cell viability was not affected compared to that of the control group (Figure 5A). In addition, the cells were cultured in HEM- or EA-free medium for 24 h, and then half of the medium was replaced with conditioned medium (CM) derived from the medium of LPS-treated BV-2 cells for 24 h. Compared to the control group, cell viability was significantly reduced by 40% among cells treated with CM (Figure 5A). Moreover, pretreatment with HEM and EA overcame the CM-induced cytotoxicity in differentiated N2a cells, and cell viability was significantly increased by 45% and 43%, respectively, compared with cells cultured in the absence of HEM or EA. However, the cells were cultured in HEM- and EA-free medium for 24 h, and then half of the medium was replaced with CM containing HEM and EA for 24 h. The results showed that cell viability was significantly reduced in comparison with the control group (Figure 5A). Therefore, we speculated that both HEM and EA directly ameliorated microglia-induced neurotoxicity.

Tyrosine hydroxylase (TH) is a standard marker for identifying dopaminergic neurons [24]. The expression of TH in differentiated N2a cells was not affected by HEM or EA treatment (Figure 5B,C). However, CM treatment significantly reduced the expression of TH in differentiated N2a cells. Furthermore, cell medium containing HEM or EA significantly improved the CM-induced reduced expression of TH in differentiated N2a cells (Figure 5B,C). Thus, pretreatment with HEM or EA could provide neuroprotection against activated microglia-induced neuronal damage.

**Figure 6:**
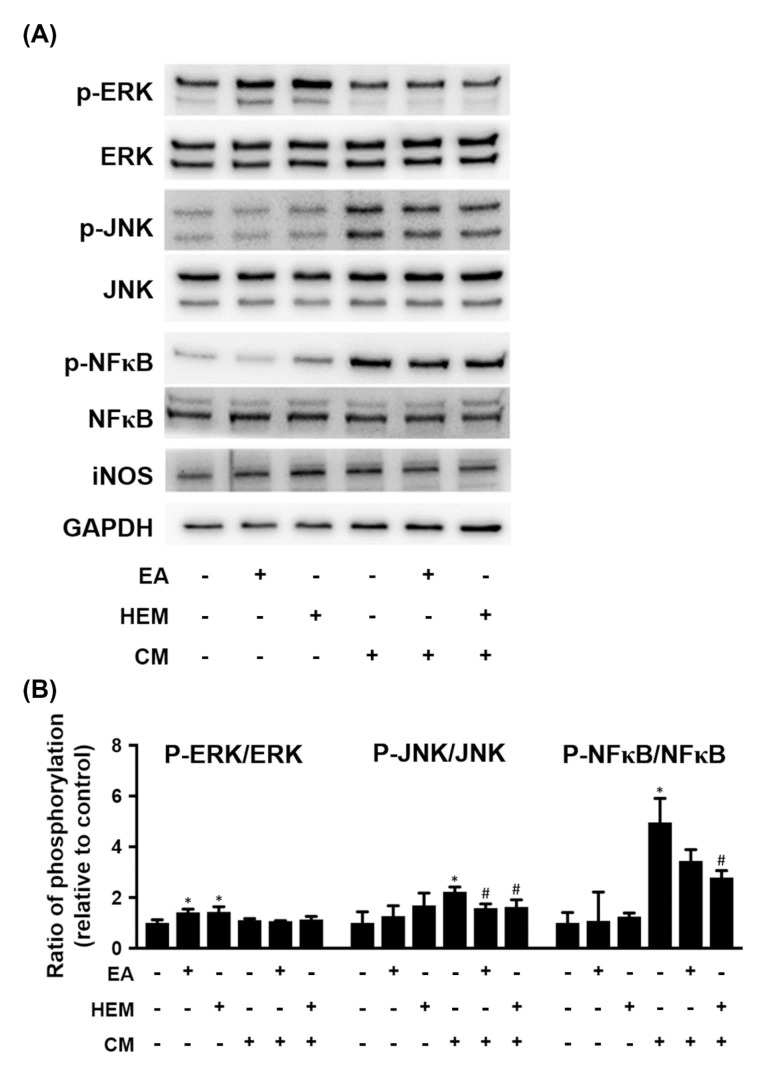
Effect of EA or HEM on signaling molecules from microglia-mediated neurotoxicity. The culture conditions of the N2a cells are shown in Figure 7. (**A**) The protein levels of ERK 1/2, JNK, NF-κB and iNOS were detected in N2a cells by western blot (*n* = 3). GAPDH was used as a loading control. (**B**) Quantitative densitometry was used to calculate the phosphorylation ratios of p-ERK to ERK, p-JNK to JNK and p-NF-κB to NF-κB, which were normalized to the control. The relative density was estimated by NIH ImageJ densitometric analysis. The results are expressed as the mean ± SD. * *p* < 0.05 compared to the control group. ^#^ *p* < 0.05 compared to the BV-2 conditioned medium treatment group.

Furthermore, we investigated the potential molecules involved in HEM- or EA-ameliorated microglia-induced differentiated N2a cell damage. HEM or EA treatment increased the ratio of pERK to ERK in differentiated N2a cells cultured in CM-free medium (Figure 6). In addition, CM treatment increased the ratio of p-JNK to JNK and P-NF-κB to NF-κB in differentiated N2a cells. Pretreatment with both HEM and EA suppressed CM-induced activation of JNA and NF-κB, respectively (Figure 6). However, the expression of iNOS was not different after differentiated N2a cells were treated with EA, HEM or CM (Figure 6). Therefore, we speculated that both HEM and EA mediated the activation of MAPKs and NF-κB in differentiated N2a cells.

### 2.3. HEM/EA Alleviates Motor Dysfunction in an LPS-Induced PD Rat Model

**Figure 7:**
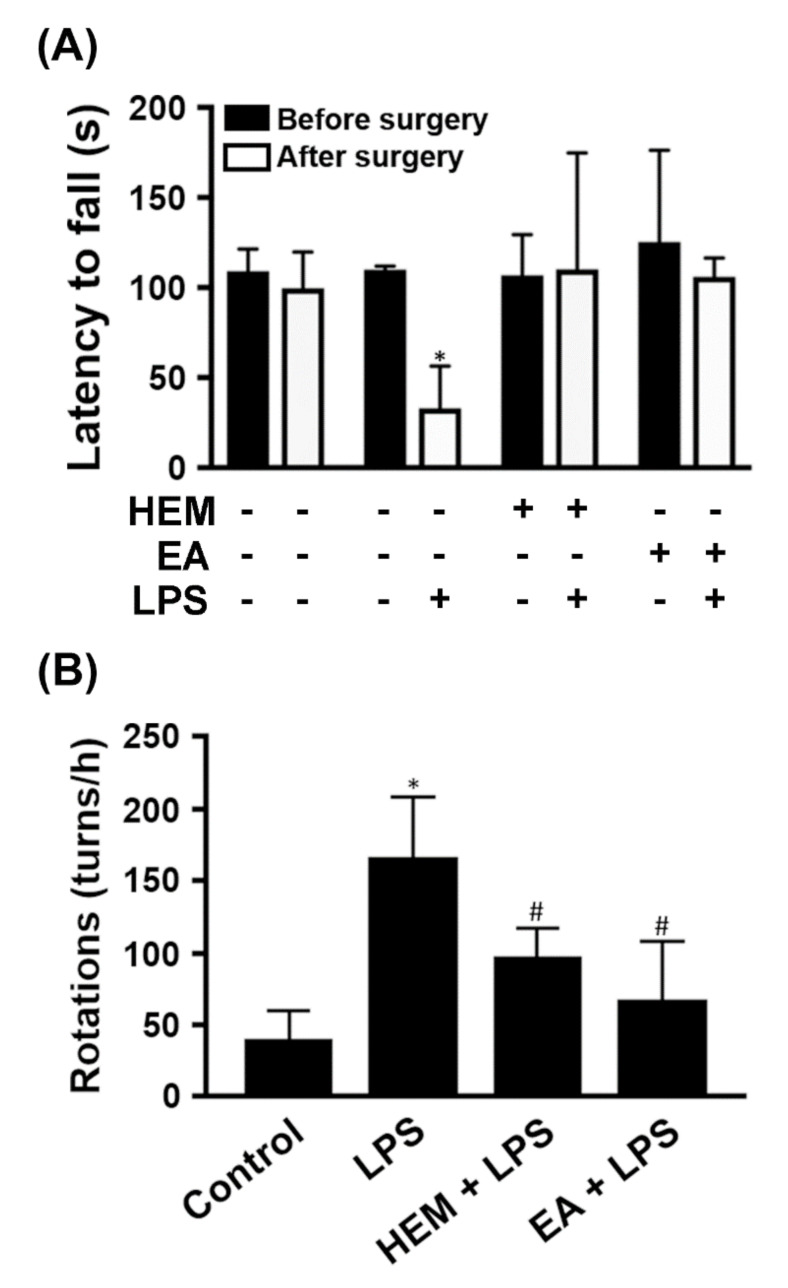
HEM or EA alleviates motor dysfunction in LPS-induced PD model rats. The rats were given vehicle, HEM (1 g/kg body weight), or EA (5 mg/kg body weight) by oral gavage once a day for 5 weeks and then they received a unilateral stereotaxic injection of LPS or PBS into the SN after the first week. (**A**) Rotarod performance analysis was performed before and after stereotaxic surgery. The amount of time the rats spent on the rotarod was measured. (**B**) After the rotarod test, the rats were tested for amphetamine-induced rotational behavior. The number of turns induced by amphetamine was recorded for 60 min. Data are presented as the mean ± SD of six rats per group. * *p* < 0.05 compared to the control group, ^#^ *p* < 0.05 compared to the LPS groups.

Our above data demonstrated that EA ameliorated the expression of proinflammatory genes in LPS-treated glia. We used a rat PD model where LPS was unilaterally injected intranigrally by stereotaxic surgery, causing a neuroinflammatory response. Rats were fed HEM (1 g/kg) or EA (5 mg/kg) by oral gavage once a day for 7 days before stereotaxic injection of LPS. The results of the rotarod performance analysis indicated that the time spent on the rotarod by HEM- or EA-treated rats compared with the control group was not different (Figure 7A). The rats had a significantly reduced time spent on the rotarod after LPS injection for 4 weeks. HEM- or EA-treated rats in the time spent on the rotarod was not different, whether with or without LPS treatment (Figure 7A). We speculated that both HEM and EA improved motor dysfunction in an LPS-injected rat.

Amphetamine, an indirect agonist of the dopamine receptor, can be used to induce rotational behavior in PD rats [25]. Therefore, administration of amphetamine induced abnormal contralateral rotations in the PD model rats [26]. After the rotarod test, the rats were injected with amphetamine, and then the rotation test was performed. LPS-injected rats had a significantly increased number of turns compared with the control group (Figure 7B). HEM- or EA-treatment significantly attenuated the LPS-induced increase in turn numbers, which were reduced by 42% and 64%, respectively (Figure 7B). Based on these data, both HEM and EA exert a neuroprotective effect on motor dysfunction in rats with LPS-induced neuronal damage.

### 2.4. HEM/EA Downregulates Proinflammatory Mediators in the Midbrain of an LPS-Induced PD Rat Model

**Figure 8:**
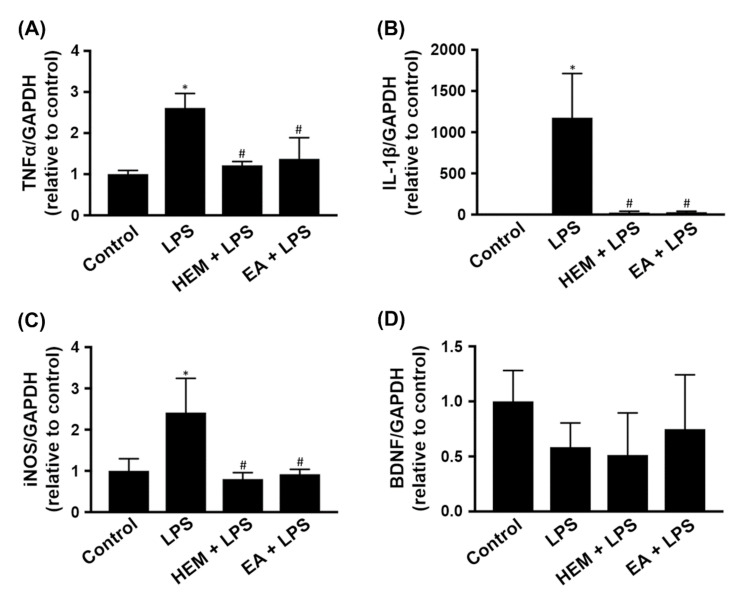
HEM or EA treatment downregulates pro-neuroinflammatory mediators in the midbrain of LPS-induced PD model rats. Rats were sacrificed after the amphetamine-induced rotation test (as shown in Figure 1). (**A**–**D**) Real-time RT-PCR analysis of proinflammatory cytokine (TNF-α and IL-1β), anti-neuroinflammatory factor (BDNF) and proinflammatory enzyme (iNOS) expression in the midbrains of rats. The data are expressed as fold changes relative to the control rats. The results are expressed as the mean ± SD. * *p* < 0.05 compared to the control group, ^#^ *p* < 0.05 compared to the LPS groups.

We further investigated the gene expression of proinflammatory mediators in the midbrain after the amphetamine-induced rotation test. Compared to the control group, LPS treatment significantly increased TNF-α, IL-1β and iNOS gene expression in the midbrain (Figure 8A,B,D). HEM or EA treatment significantly attenuated the LPS-induced increase in the gene expression of TNF-α, IL-1β and iNOS in the midbrain (Figure 8A,B,D). Based on previous results, we speculated that both HEM and EA mediated LPS-induced activation of microglia and astrocytes leading to the reduction of iNOS and TNF-α expression, respectively (Figure 2 and Figure 4). However, the gene expression of BDNF in the midbrain was not affected in rats fed with or without HEM or EA and injected with LPS or PBS (Figure 8C). Therefore, we speculated that the neuroprotective effect of HEM and EA on LPS-induced neurotoxicity might occur through downregulation of the gene expression of proinflammatory factors.

## 3. Discussion

### 3.1. EA Modulates LPS-Induced Expression of Proinflammatory Factors in Glial Cells

The major finding of this study was the different effects of EA on LPS-induced glial cells, which significantly decreased TNF-α expression in CTX TNA2 cells but not in BV-2 cells (Figure 1 and Figure 4). In addition, EA reduced both iNOS expression and NO production in BV-2 cells treated with LPS and IFN-α (Figure 2) and did not alter the expression of iNOS in CTX TNA2 cells (Figure 3). The expression of iNOS was significantly increased in CTX TNA2 cells treated with 250 ng/mL LPS (Figure 3), but iNOS was not increased in BV-2 cells treated with 250 ng/mL or 500 ng/mL LPS (data not shown). Moreover, BV-2 cells were treated with 500 ng/mL LPS, and 50 ng/mL IFN-α induced a significant increase in iNOS expression. Perhaps the concentration of LPS was too high, resulting in only the highest concentration of EA (20 μM) ameliorating iNOS expression in BV-2 cells (Figure 2).

LPS is an agonist of Toll-like receptor-4 (TLR-4) that induces proinflammatory signaling cascades causing acute sepsis or chronic inflammatory disorders [26]. LPS-induced BV-2 cells switch to a proinflammatory phenotype (the M1 phenotype) [27]. In addition, LPS-stimulated astrocytes display a proinflammatory phenotype (A1 phenotype) and secrete inflammatory factors [28]. Our data indicated that LPS-treated glial cells, BV-2 cells and CTX TNA2 cells, significantly increased the expression of TNF-α which is a marker of proinflammatory phenotype for glial cells. EA pretreatment prevented LPS-induced TNF-α expression in CTX TNA2 cells (Figure 4). iNOS is another marker of proinflammatory phenotype for glial cells. EA pretreatment prevented LPS and IFN-γ induced iNOS expression in BV-2 cells (Figure 2). Therefore, EA-modulated LPS-induced proinflammatory responses vary by the type of glial cell.

Furthermore, the results from the in vivo study indicated that administering both EA and HEM significantly reduced the expression of the proinflammatory factors TNF-α, IL-1β and iNOS in the midbrains of rats with SN injection of LPS. Herein, EA suppressed the LPS-induced inflammatory response in the midbrain, i.e., EA ameliorated the proinflammatory phenotype of glial cells. Based on the data shown in Figure 8, we speculated that LPS-induced expression of lower BDNF and higher TNF-α, IL-1β and iNOS in the midbrain compared with the control group was related to an increased proinflammatory phenotype of astrocytes. However, the expression of BDNF in the midbrain was not affected in the rats with or without both EA and HEM. We speculated that neither EA nor HEM changed astrocytes into an anti-inflammatory phenotype (A2 phenotype). In addition to BDNF, other neurotrophic factors, e.g., NGF and NT3, should be investigated in the future to understand better whether EA modulates the neuroinflammatory response in PD.

### 3.2. EA Protects Dopaminergic Cells against Activated Microglia-Induced Neuronal Cell Death

Neuroinflammation plays a role in the progressive loss of dopaminergic neurons in the SN and is a prominent part of the pathogenesis of PD. The results showed that LPS induced the activation of BV-2 cells (Figure 1). The culture medium of LPS-treated BV-2 cells induced significant death of differentiated N2a cells and reduced TH expression in these cells, but pretreatment with both EA and HEM ameliorated conditioned medium-induced cell death (Figure 5).

LPS injected into the substantia nigra (SN) of rats is an established PD animal model, and LPS induces a strong microglial reaction causing a neuroinflammatory response leading to dopaminergic neuronal loss [29]. The results of the in vivo studies, rotarod performance tests and amphetamine-induced rotational behavior tests indicated that rats fed both EA and HEM had ameliorated LPS-induced motor dysfunction (Figure 7). Both EA and HEM protect dopaminergic neurons from LPS-induced neuroinflammation. In addition, the protective effect of both EA and HEM seemed to act directly on N2a cells. CM containing EA or HEA did not possess a protective effect in N2a cells (Figure 5). Therefore, we speculate that both EA and HEM have preventive effects on the neuroinflammation-induced death of dopaminergic neurons.

As shown in Figure 6, both EA and HEM inhibited CM-induced JNK and NF-κB activation. JNK signaling is associated with apoptosis in neurons [30] and is critical for the pathological cell death observed in Parkinson’s disease [31]. NF-κB is a therapeutic target in PD and plays an important role in the activation and regulation of neuroinflammation [32]. In addition, the results showed that both EA and HEM enhanced ERK activation in differentiated N2a cells, but CM treatment did not affect ERK activation (Figure 6). ERK1/2 is believed to promote cell growth and differentiation. Therefore, we speculate that CM induces N2a cell death via both the JNK and NF-κB signaling pathways but not ERK. It is also necessary to investigate the underlying mechanisms by which both EA and HEM protect dopaminergic neurons from inflammatory factor-induced cell death in the future.

Many studies have provided evidence that EA or HEM have neuroprotection effects in vivo. EA increases the NGF content in the central nervous system of rats [33]. EA-enriched HEM induces an antidepressive effect in depressed mice challenged by repeated restraint stress [18], improves learning and memory and delays degenerative aging in senescence-accelerated mouse prone 8 mice [34]. HE ethanol extract prevents neuronal death after seizures in mice with pilocarpine-induced status epilepticus [35], improves recognition memory and induces neurogenesis in frail mice during aging [36]. Both EA and HEM protect neurons from ischemic injury in a stroke animal model [19], ameliorate Alzheimer’s disease-related pathologies in APPswe/PS1dE9 transgenic mice [20,21], improve spatial learning deficits in obese-aging mice [37] and protect dopaminergic neurons from MPP+-induced neurotoxicity in an MPTP-induced mouse model of PD [22]. Our results indicated that both EA and HEM inhibited the expression of proinflammatory factors in the midbrain and ameliorated motor dysfunction after the intranigral injection of an LPS-induced PD animal model.

In view of the above studies, both EA and HEM have shown neuroprotective effects in different animal models. In the animal studies mentioned above, the feeding dose of HEM was less than 1 g/kg/body weight/day. Li et al. indicated a safe dose of EA-enriched HEM of up to 3 g/kg body weight/day in toxicology testing in a 28-day oral feeding study in Sprague–Dawley rats [38]. Therefore, we speculate that HEM could be a nutritional supplement for preventing PD. Moreover, EA could be an adjuvant agent in the prevention and treatment of PD, but the toxicological safety of EA needs to be assessed in the future.

In summary, EA pretreatment inhibited the expression of proinflammatory factors involved in the activation of glial cells, i.e., it reduced the expression of TNF-α and iNOS in CTX TNA2 cells treated with LPS and in BV-2 cells treated with LPS and IFN-α, respectively. Both EA and HEM protected differentiated N2a cells from LPS-treated BV-2 conditioned medium-induced cell death by suppressing the activation of both JNK and NF-κB. In vivo, both EA and HEM ameliorated motor dysfunction from the LPS-induced neuroinflammatory response in a PD animal model. Our data revealed that EA pretreatment ameliorates LPS-stimulated microglia-mediated neuroinflammation, resulting in the protection of dopaminergic neurons and the prevention of motor dysfunction both in vitro and in vivo.

## 4. Materials and Methods

### 4.1. Chemicals and Reagents

Antibodies against phosphorylated p-ERK, p-JNK, p-NF-κB, ERK, JNK and NF-κB were obtained from Cell Signaling Technology (Beverly, MA, USA). Rabbit anti-tyrosine hydroxylase (TH) antibodies were purchased from Novus Bio. Co. (Littleton, CO, USA). LPS (*Escherichia coli*, serotype 0111:B4) was purchased from Sigma-Aldrich Co. (St. Louis, MO, USA). Rompun 2% injection was purchased from Bayer (Yongin, Gyeonggi-do, Korea). Zoletil 50 was purchased from VIRBAC (Virbac, Carros, France). All cell culture reagents were purchased from Invitrogen (Carlsbad, CA, USA). All other chemicals were reagent grade or higher and were purchased from Sigma-Aldrich Co (St. Louis, MO, USA). Nitrate/nitrite colorimetric assay kits were obtained from Cayman Chemical (780001) (Ann Arbor, MI, USA). *H. erinaceus* sample preparation and the identification of erinacine A were as described in our previous publication [39].

### 4.2. Cell Culture

BV-2 cells were cultured with Dulbecco’s modified Eagle’s medium (DMEM) supplemented with 2.2 g sodium bicarbonate, 4.8 g 4-(2-hydroxyethyl)-1-piperazineethanesulfonic acid (HEPES) buffer adjusted to pH 7.2–7.4 and containing 1% (*v*/*v*) antibiotics, 2 mM L-glutamine, and 10% (*v*/*v*) fetal bovine serum (FBS) (Gibco, Grand Island, NY, USA). BV-2 cells were cultured in plates at a density of 10^5^ cells/cm^2^ at 37 °C in a humidified atmosphere of 5% CO_2_ and 95% air. Activation was induced by lipopolysaccharide (LPS). The shape of the BV-2 cells was round at rest and an irregular spindle after they were activated by LPS [32].

The immortalized astrocyte cell line (CTX TNA2) was kindly provided by Prof. His-Lung Hsieh (Chang Gung University of Science and Technologies, Taoyuan, Taiwan). The cells were fed every 2–3 days with DMEM, pH 7.2, containing 1% (*v*/*v*) antibiotics, 2 mM L-glutamine, and 10% (*v*/*v*) FBS. Confluent cultures were subcultured with a 0.1% trypsin–EDTA solution.

Both BV-2 cells and CTX TNA2 were treated with the indicated concentration of EA (0, 5, 10 or 20 µM) for 15 min and then stimulated with or without LPS (250 ng/mL) for the indicated times (6 h, 12 h or 24 h). In addition, BV-2 cells were treated with the indicated concentration of EA (0, 5, 10 or 20 µM) for 15 min and then stimulated with or without LPS (500 ng/mL) and IFN-γ (50 ng/mL) for 24 h.

Mouse neuro-2a (N2a) cells were cultured in DMEM with high glucose (4.5 g/L), stable glutamine, and sodium pyruvate supplemented with 10% (*v*/*v*) FBS and containing 1% (*v*/*v*) antibiotics. To obtain N2a differentiated neurons, the medium was changed to DMEM + 0.5% FBS with dbcAMP (1 mM) for 5 days. N2a cells are widely used to study neuronal differentiation and neurite outgrowth.

The differentiated N2a cells were treated with or without EA (10 µM) or HEM (1 mg/mL) for 48 h or 24 h, half of the medium was removed, and the same amount of conditioned medium was added for 24 h. Conditioned medium (CM) was derived from the culture medium of BV-2 cells with 250 ng/mL LPS treatment for 24 h.

### 4.3. Animal Preparation and Surgery

All animal studies were approved by the Institutional Animal Care and Use Committee (IACUC) of Chung Yuan Christian University under project number 106,011 (accessed on 10 August 2017). Male Sprague–Dawley (SD) rats were purchased from BioLASCO Taiwan Co., Ltd. (Taipei, Taiwan). The weight of the rats was approximately 250 to 300 g, and the rats were given ad libitum access to food and water and kept on a 12/12 h light/dark cycle during the study.

The rats were randomly divided into four groups: the PBS-injected group (Control), the LPS-injected group followed by vehicle pretreatment (LPS group), the LPS-injected group followed by pretreatment with HEM or EA, the HEM + LPS group, and the EA + LPS group. Rats in the HEM or EA pretreatment groups were treated by daily oral gavage starting 1 week before the surgery and continuing for 5 weeks. LPS (or PBS) was injected unilaterally into the SN as previously described [32]. Briefly, the rats were anesthetized intraperitoneally with 0.06 mL/10 g of Zoletil 50 (50 mg/mL) diluted 5× (1 mL Zoletil in 0.1 mL Rompun 2% and 3.9 mL normal saline) and mounted in a stereotactic frame equipped with a rat adaptor. The fur on the head of the rats was shaved off, their skin was disinfected with povidone-iodine, and the skull was exposed. PBS or 3 μL LPS (5 μg/μL) was injected into the right substantia nigra (anteroposterior (AP), 5.3 mm, mediolateral (ML), 2.3 mm and dorsoventral (DV), 7.7 mm from bregma). Infusions were made at a rate of 1 µL/min for LPS or PBS. Then, the needle was left in situ for 5 min to avoid reflux along the injection track. The skin was sutured. Four weeks after LPS injection, all animals underwent behavioral evaluations.

### 4.4. Real-Time Quantitative Reverse-Transcription Polymerase Chain Reaction (qRT-PCR)

RNA isolation from cells and SN tissue samples was performed using TRIzol reagent according to the manufacturer’s protocol (Invitrogen, Carlsbad, CA, USA), and then 1 µg of total isolated RNA was used for reverse transcription with SuperScript III Reverse Transcriptase (Invitrogen) using random primers. qRT-PCR was performed using FastStart Universal SYBR Green Master Mix (Rox) (Roche AG, Mannheim, Germany). Thermocycling was performed with a 7300 Real Time 40 PCR system (Applied Biosystems). Conditions for qRT-PCR included initial denaturation at 94 °C for 1 min, followed by 98 °C for 15 s and 60 °C for 40 cycles. The primers used in this study were as follows (Table 1):

### 4.5. Locomotor Activity Measurements

A rotarod test was performed to study muscular coordination. It is also a classic method to evaluate behavioral dysfunction in PD model rats. Before the experimental surgery, all rats underwent a 2-day training program on the rotarod. The rats were placed on the rod and its speed gradually accelerated from 0.35 to 3.5 m/min. The time latency to fall from the rotarod was recorded. A baseline trial was conducted before the surgery. Fourth weeks after the surgery, the rats were tested again.

### 4.6. Rotational Behavior Assay

Fourth weeks after the LPS injection, the rats were given amphetamine (2 mg/kg, i.p.) in order to assess the lesion severity. For that, the rats were placed in individual plastic hemispherical bowls and injected with amphetamine following a short period of acclimatization. Turns were enumerated 30 min postinjection and recorded over a 60 min observation period [41].

### 4.7. Western Blot Analysis

The protein levels of TH, iNOS, p-NF-κB, NF-κB, p-ERK1/2, ERK1/2, p-JNK and JNK were monitored in whole-cell lysates or SN tissues. Briefly, after each treatment, cells or midbrain tissue were lysed and centrifuged at 16,000× *g* for 30 min at 4 °C, and then the supernatant was collected. Aliquots of 30 µg of protein were mixed with SDS–PAGE sample buffer, electrophoretically separated and transferred onto a PVDF membrane. After blocking nonspecific binding, the blots were then sequentially hybridized with the primary and secondary antibodies. Antibodies against phosphorylated (p)-ERK (#9101, 1:1000), ERK (#9102, 1:1000, (p)-JNK (#9251, 1:1000, JNK (#9252, 1:1000), (p)-NF-κB (#3033, 1:1000), NF-κB (#4764, 1:1000) and GAPDH (#2118, 1:5000) were obtained from Cell Signaling Technology. Rabbit anti-tyrosine hydroxylase (TH) antibodies were purchased from Novus Bio. Co. (NB300-109; 1:1000; Littleton, CO, USA). Secondary antibodies (1:4000; Jackson ImmunoResearch Laboratories, West Grove, PA, USA). Finally, the blots were developed with enhanced chemiluminescence detection reagents (Amersham Biosciences). ImageJ software (NIH, Bethesda, MD, USA) was used to quantify the relative fold change compared to that of the control.

### 4.8. Nitric Oxide Release

BV-2 cells were treated with EA for 15 min and then stimulated with LPS and IFN-γ for 24 hr. Next, we collected 50 μL of culture media from each culture condition and transferred them into 96-well plates. Fifty microliters of Griess reagent was used to evaluate the NO concentration in the cells and it was added to the different wells. The plates were protected from light and incubated at 25 °C for 20 min. An ELISA reader assessed the optic density (OD) values at 540 nm wavelength to determine the concentration of NO released by the cells [40].

### 4.9. TNF-α Assessment

We cultured BV-2 cells or CTX TNA2 in 6-well plates. The cells were then treated with EA and incubated for 15 min. We induced inflammation by treating the cells with LPS and incubating them for 6, 12 or 24 h. The culture media were collected for TNF-α secretion assessment using a TNF-α mouse ELISA kit (R&D Systems) [40].

### 4.10. Statistical Analyses

The data are presented as the mean ± SD and were analyzed using SigmaPlot (version 11, Systat Software, Inc., San Jose, CA, USA). The data from the Rotarod performance analysis were analyzed by repeated measures analysis of variance followed by paired *t* test. Other significant differences between two groups (e.g., control and LPS, LPS and EA, or LPS and HEM) were analyzed by one-way ANOVA with post-hoc Tukey HSD test. * *p* < 0.05 indicated a statistically significant difference compared to the control group, while ^#^ *p* < 0.05 indicated a statistically significant difference compared to the LPS group.

## Figures and Tables

**Table 1:** Primer sequences used for qRT-PCR analysis [40,41].

Gene Name	Primer Sequence
BDNF	Forward: 5′-GGTCACAGCGGCAGATAAAAAGA-3′ Reverse: 5′-TTCGGCATTGCGAGTTCCA-3′
GAPDH	Forward: 5′-GCAAGAGAGAGGCCCTCA-3′ Reverse: 5′-TGTGAGGGAGATGCTCAGTG-5′
IL-1β	Forward: 5′-GAGCACCTTCTTTTCCTTCATCT-3′ Reverse: 5′-GATATTCTGTCCATTGAGGTGGA-3′
iNOS	Forward: 5′-GGTATGAAATGGCAAATCGG-3′ Reverse: 5′-CGGACCATCTCCTGCATT-3′
TNF-α	Forward: 5′-CTCACACTCAGATCATCTTCT-3′ Reverse: 5′-GGTATGAAATGGCAAATCGG-3′

The GAPDH gene was used as a reference housekeeping gene for normalization.

## Data Availability

The data presented in this study are available on request from the corresponding author.
